# Effect of 11 Weeks of Physical Exercise on Physical Fitness and Executive Functions in Children

**DOI:** 10.3390/children10030485

**Published:** 2023-03-01

**Authors:** Mingyang Zhang, Hanna Garnier, Guoping Qian, Shunchang Li

**Affiliations:** 1 Department of Physical Education, Chengdu Sport University, Chengdu 610041, China; 2 Faculty of Physical Culture, Gdansk University of Physical Education and Sport, 80-336 Gdansk, Poland; 3 Department of Surgery and Urology for Children and Adolescents, Medical University of Gdansk, 80-210 Gdansk, Poland; 4 Institute of Sports Medicine and Health, Chengdu Sport University, Chengdu 610041, China

**Keywords:** physical exercise, physical fitness, executive functions, children

## Abstract

Object: The aim of our study was to evaluate and compare the effects of physical exercise interventions on physical fitness and executive functions in children. Methods: Six-year-old children participated in the study and were randomly divided into physical exercise group (PE group, *n* = 43) and control group (C group, *n* = 46). The children in the PE group participated in a physical exercise program for 45 min daily, four days a week for 11 weeks. The children in the C group continued with their usual routines. Then, all the children were tested before and after the experiment for body composition (height, weight, BMI), physical fitness (20-m shuttle run test, standing long jump test, grip strength test, 4 × 10 m shuttle run test and sit and reach tests), and executive functions test (animal go/no-go task, working memory span task, simple reaction test and flexible item selection task) before and after the 11-week period. Results: The 11 weeks of physical exercise did not significantly affect the body composition of the children (*p* > 0.05). The physical fitness and executive functions test results showed that 11 weeks of physical exercise interventions improves physical fitness (cardiopulmonary fitness, muscle strength, speed sensitivity and flexibility quality) and executive functions parameters (inhibitory control, working memory, the reaction time, and cognitive flexibility) in children (*p* < 0.05, *p* < 0.01). Conclusion: 11 weeks of physical exercise can improve the physical fitness and executive functions of six-year-old children.

## 1. Introduction

Physical fitness (PF) is defined as the body’s condition related to lifestyle that includes strength, speed sensitivity, and cardiopulmonary function [1]. According to a PF survey in China, the trend of continuous decline in children’s PF has not changed in recent years [2]. Moreover, evidence indicates that PF in childhood moderately affects PF in adulthood [3]. A growing body of evidence focuses not only on the PF of healthy children and those with disease, but also on the executive functions (EFs) of these children [4,5]. EFs are represented by a set of higher-order cognition of the prefrontal cortex brain region for goal-directed thought and action [6]. Evidence suggests that EFs have significant effects, including attention, reaction, and spatial memory, on children [7,8]. The development of EFs is associated with the maturation of the prefrontal cortex during childhood [7], and prefrontal cortical thickness mediates the association between cortisol reactivity and EFs in children [9]. Inversely, children exhibit partially compromised EFs, which may be partly explained by the reduced cortical thickness in the prefrontal cortex [10]. Overall, EFs play a pivotal role in children’s brain health and school performance, and the importance of EFs in children begins in early childhood and continues throughout development. Based on the above, there has been a strong call to improve PF and EFs among children and adolescents for global health and social development. Therefore, it is of great importance to develop effective measures to improve children’s PF and EFs.

Physical exercise has many physical and mental health benefits, such as helping an individual to maintain a healthy body weight by maintaining energy balance, to enhance the endurance performance of the muscles, and to relieve anxious or depressive states. Neurobiology research has shown that physical exercise, with almost no side effects, can be beneficial to children. Several studies have emphasized the significant contribution of physical exercise to PF. For example, the study performed by Neil et al. [11] showed that school-based physical exercise interventions may improve PF in children and adolescents. In addition, physical exercise can directly improve children’s EFs and indirectly enhance EFs mediated by physical fitness, such as limb strength, speed sensitivity, agility, balance, and flexibility [12]. Moreover, the frequency and duration of physical activity, as well as physical exercise intensity are consistently and favorably associated with multiple PF indicators [13]. Accumulating evidence has shown that physical exercise interventions in children not only improves their PF, but also improves brain health, academic performance, and EFs. A meta-analysis reported the positive effect of physical exercise interventions on EFs in children, such as inhibition/interference control, working memory cognitive flexibility, and planning [14,15]. Mehren’s [16] study was the first to show that different exercise intensities (moderate intensity vs. high intensity) have different effects on EFs as measured by functional magnetic resonance imaging (fMRI). Furthermore, children are in a critical period of basic motor development, and there are closed interrelation between motor ability, cognitive function development and prefrontal cortex [17]. It is important to note that EFs in children are malleable and are influenced by the plasticity of the cerebral cortex structure [18]. Such evidence directly highlights the positive effect of physical exercise on EFs. The literature on the intervention of physical exercise on EFs in children is abundant, but current research on the comparative study of pre-test and post-test results as well as intragroup difference is lacking. Therefore, it is necessary to conduct comparative research to explore the effects of physical exercise on EFs in children.

In order to explore the effect of 11-week physical exercise interventions on PF and EFs in children, we intervened in the form of 11 weeks of physical exercise on children to observe the effect of improvement on the PF and EFs in six-year-old children. Therefore, for the test of this study, we adopted repeated measurements to observe the improvement of the PF and EFs in children, which were measured once before the start of the training and once at the end (at 11 weeks). Our study hypothesized that physical exercise interventions would improve PF and EFs in children. Based on this, we designed an experiment on the intervention effect of physical exercise on children.

## 2. Materials and Methods

### 2.1. Participants

**Figure 1:**
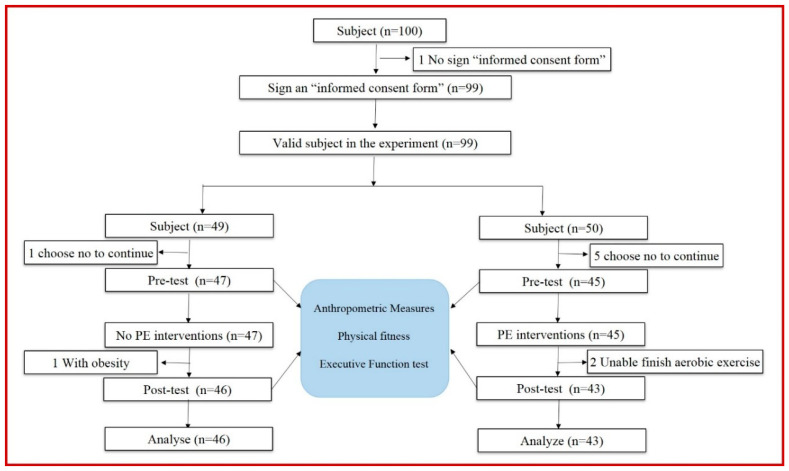
Participant flowchart across the study.

The participants in our study were first grade elementary school students in Beijing, China. We excluded children with medical conditions that affect body composition, PF, and EFs. This study adopted a randomized controlled design. A total of 100 healthy right-handed children (Boys = 50; Girls = 50; six-year-olds) were enrolled in our study before the experiment and randomly divided into a control group (C group, *n* = 49) and physical exercise group (PE group, *n* = 50). Finally, a total of 89 students completed the whole experiment, and their data were considered effective samples (C group, *n* = 46; PE group, *n* = 43; Figure 1). We ensured that the sex ratios of the two groups were as equal as possible during participant grouping. All the children were tested before and after the experiment based on four PF indicators and four EFs task parameters. The four PF indicators in this study included cardiopulmonary fitness (20-m shuttle run test), muscle strength (standing long jump test and grip strength test), speed sensitivity (4 × 10 m shuttle run test), and flexibility quality (sit and reach). The four EFs task parameters assessed through EF touch testing and the Psykey psychological system included the animal go/no-go task, working memory span task, simple reaction test, and flexible item selection task. In addition, we obtained informed consent from the children and their parents/guardians before their participation in the study. All experimental procedures and materials were reviewed and approved by the institutional review board of Capital University of Physical Education and Sports.

### 2.2. Physical Exercise Interventions

The physical exercise protocol was developed based on previous literatures [19,20]. In this experiment, the protocol was implemented in the school setting, and aimed to increase physical activity in children. All the children in the PE group participated in the physical exercise program four days a week for 11 weeks. Two teachers (one man and one woman) guided the children through the exercises during the experiment. 

**Figure 2:**
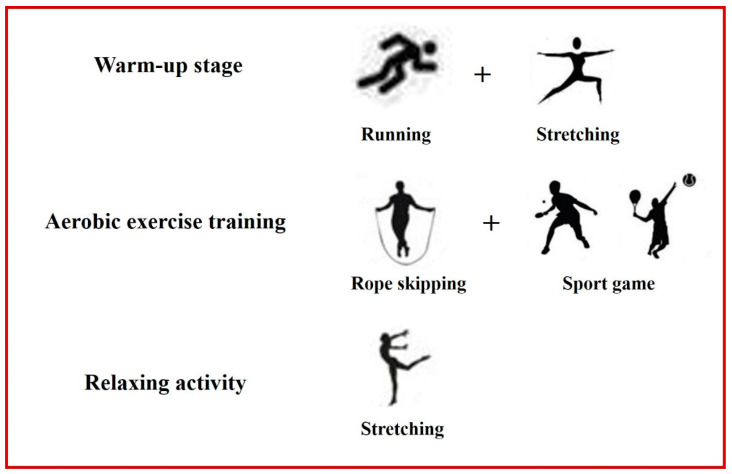
Exercise program in this experiment.

The exercise program consisted of three stages including the warm-up, aerobic exercise, and relaxing activity stages. The warm-up stage lasted for 5 min and included activities, such as running and stretching. Subsequently, the children participated in a 30-min aerobic exercise training, including jumping rope and sport games (60%–70% of maximum heart rate). Finally, the children participated in a relaxation activity, such as stretching exercises of the upper and lower limb muscles for 5 min (Figure 2). To increase motivation and adherence to the protocol, all exercise sessions were selected based on ease of comprehension, enjoyment, and safety. In addition, all the three stages of the physical exercise intervention program were included in a school-based exercise program with guidance from a physical therapist who works in a hospital to optimize physical health.

### 2.3. Body Composition and Sedentary Behavior

In this study, body composition and sedentary behavior were assessed for each child. Height (cm) and weight (kg) were measured using a Holtain stadiometer and digitized weighing scales, respectively, with the participants lightly dressed and barefooted. Body mass index (BMI) was calculated by dividing body weight in kilograms by height squared in meters (kg/m^2^).

### 2.4. PF Test

The detailed procedure for the PF test has been previously described in detail [21,22]. The PF test in this study included cardiopulmonary fitness (20-m shuttle run test), muscle strength (standing long jump and grip strength tests), speed sensitivity (4 × 10 m shuttle run test), and flexibility quality (sit and reach). All the PF tests were conducted by qualified personnel during school hours (9 am–4 pm), and all participants performed routine warm-up exercises before the tests. The 4 × 10 m shuttle run test was always the last test performed.

The 20-m shuttle run test was performed to assess cardiopulmonary fitness. The participants stood 0.3 m in front of the starting line. This test required participants to run back and forth between two lines set 20 m apart. The initial running speed was 6.5 km/h, which was increased by 0.5 km/h. Children were encouraged to exhibit their best performance during tests. This test ended when the child demonstrated fatigue. The final test score was recorded as the number of laps completed. 

Muscle strength was assessed using two different tests: (1) The standing long jump test was used to assess lower limb muscle strength. The jump distance for this test was measured in cm. Children jumped a distance with both feet simultaneously off the ground on an international standard playground. They performed three jumps with 30 s rest intervals between attempts. The best distance among the three jumps was used for the analysis. (2) Grip strength test was used to evaluate upper limb muscular strength, which was measured using a handgrip dynamometer (TKK 5001, gripA, Takei, Tokyo). The children stood upright with their shoulders in a neutral position, and arms at their side. Then, the children were instructed to use each hand to squeeze the handle of the dynamometer with maximum effort for 3 s. Each hand was tested three times, alternating the hands between trials, with 30 s rests intervals between measurements on the same hand. Absolute grip strength was calculated as the highest registered value for each hand and is expressed in kilograms (to the nearest 0.1 kg).

The 4 × 10 m shuttle run test was used to assess speed sensitivity. The children run back and forth four times along a 10-m track at maximum speed. The children performed this test twice, and the best result (minimum time in seconds) was recorded for the analysis.

Flexibility quality was assessed using the sit and reach test. The children sat on the floor with their head, back, and hips in contact with the wall and both legs fully extended. They stretched out their upper limbs as far as possible to touch their toes. The test was performed twice, and the better result was recorded.

### 2.5. EFs Test

All the EFs tests were conducted in the classrooms, and the children performed these tests before and after the 11-weeks physical exercise interventions. All the test tasks were performed in sequence according to the procedure. Four main EFs parameters, including animal go/no-go task, working memory span task, simple reaction test, and flexible item selection task, were measured using EF touch testing and Psykey psychological system. The data obtained from the children for the EF tests were considered not valid if the procedure was terminated due to child fatigue/stress or due to experimental error.

It is worth noting that all the participants were eligible for the hand grip strength component of this test. Individuals were excluded if they had undergone hand surgery within the previous 3 months prior to the study and if they had any pain, or stiffness in their right hand (e.g., arthritis or tendinitis) in the previous 7 days prior to the study. Details of the EF tests are as follows:

The animal go/no-go task is a commonly used test to evaluate inhibitory control [23]. The task involves the random presentation of seven animals above a green button. If the animal is not a pig, the children are required to quickly press the green button. When a pig is presented, no button is pressed. Only one animal was presented at a time for a maximum of 3 s, and the process was 40 times. The correct rate and reaction time were recorded.

The working memory span task was employed to analyze working memory as previously described [24]. First, a house was appeared on the screen, and the children were required to note and remember the information in the house, such as the name of the animal in the house and the color of the windows. Subsequently, the experimenter asked the children some information about the house. Next, an empty house appeared on the screen, and the children had to answer some questions about the house. The number of houses increased from one to three during the test.

The simple reaction test was used to evaluate the reaction time in our experiment [25]. When the stimulus of a green circle presents on the screen of a computer, the children were required to quickly press a green button as soon as possible. A total of 30 trials were performed with a 2 s interval.

The flexible item selection task was performed to assess cognitive flexibility [26]. The children were required to sort the cards by color, size, or category. Two pictures of the same category appeared on the screen, and the children had to indicate the category of the pictures. Next, when a new picture was shown on the screen, and the children had to indicate its category as with the first two images. Finally, three pictures were shown together, and the children chose the two pictures that were similar in a certain dimension.

### 2.6. Statistical Analysis

All data were presented as mean ± SD. Statistical analysis was performed by SPSS 20.0 and GraphPad Prism software. In body composition, PF test, the comparison among different groups at the same time was tested by independent-samples T test. The paired sample T test was used to compare before and after the experiment in the same group. In order to examine the effect of the physical exercise intervention, a 2 × 2 repeated measures ANOVA was used with the within factor (before/after intervention) and the between factor EXERCISE (exercise/no exercise). A statistically significant level was defined as *p* < 0.05.

## 3. Results

### 3.1. Basic Information of Children

The children’s body composition and sedentary behavior details for each group are presented in Table 1. There was no statistically significant difference between the C and PE groups before the physical exercise interventions in body composition indices, such as height, weight, BMI (*p* > 0.05). A similar phenomenon was observed after the 11-week period as no significant changes in body composition indices were observed over time in both groups. In addition, no remarkable difference of body composition index in each group with the paired sample *t*-tests (*p* > 0.05). Thus, there was no significant effect of 11 weeks physical exercise on body composition in children.

### 3.2. Effect of 11 Weeks of Physical Exercise on PF in Children

**Figure 3:**
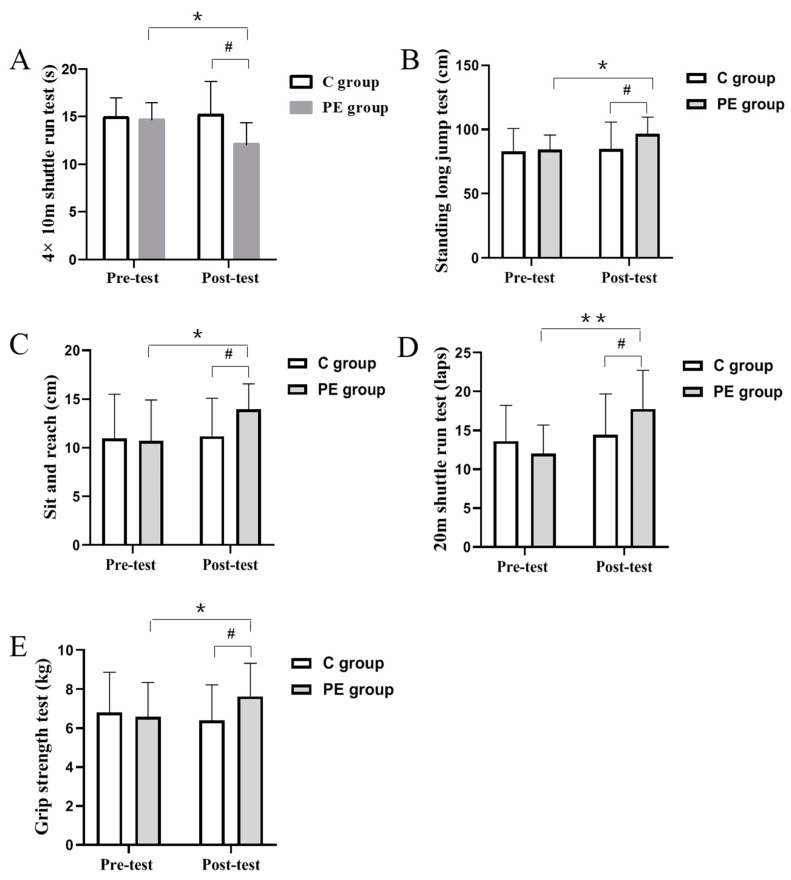
PF parameters of each group in pre-test and post-test. The results of 4 × 10 m shuttle run test (**A**), standing long jump test (**B**), sit and reach tests (**C**), 20-m shuttle run test (**D**), grip strength tes t (**E**) in each group. * *p* < 0.05, ** *p* < 0.01 vs. PE group. # *p* < 0.05 vs. C group.

Figure 3 shows the different PF parameters for each group. After 11 weeks of physical exercise interventions, the PF parameters including the 20-m shuttle run test, standing long jump test, grip strength test, 4 × 10 m shuttle run test, sit and reach tests were significantly improved in the PE group than that observed before the 11 weeks (*p* < 0.05, *p* < 0.01). However, there were no significant differences in the C group measured before and after the 11 weeks (*p* > 0.05).

In addition, pre-test results revealed no significant difference of PF parameters between the control and PE groups before physical exercise interventions, including the 20-m shuttle run test, standing long jump test, grip strength test, 4 × 10 m shuttle run test, sit and reach tests (*p* > 0.05). When differences in physical fitness were compared in children using cardiopulmonary fitness, muscle strength, speed sensitivity, flexibility quality, all variables showed a significant intergroup difference (*p* < 0.05). These data supported that 11 weeks of physical exercise interventions improve PF parameters in children.

### 3.3. Effect of 11 Weeks of Physical Exercise on EFs on Children

Before the physical exercise interventions, variance analysis was performed on each function included in the experiment, and we found that the pre-test accuracy of the inhibitory control, the working memory, the reaction time, and the cognitive flexibility were not significantly different between the two groups, indicating no difference in the EFs level of the children in both groups before the 11-week period (*p* > 0.05).

**Figure 4:**
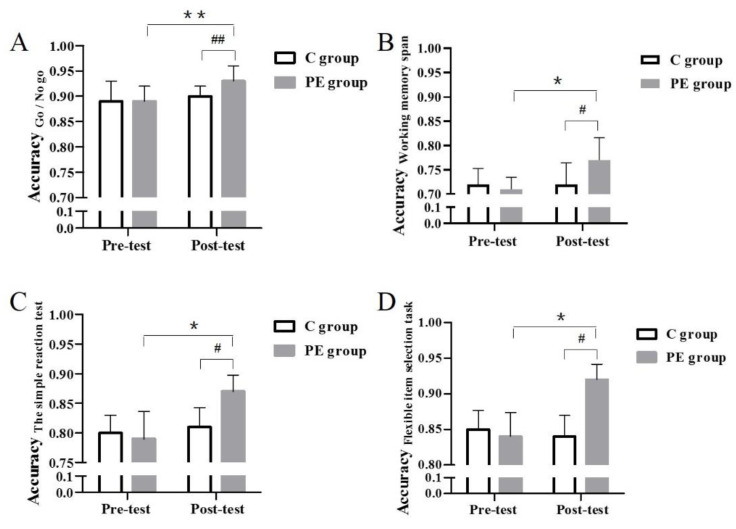
EFs accuracy of each group in pre-test and post-test. The results of accuracy in animal Go/No-go task (**A**), working memory span task (**B**), simple reaction test (**C**), flexible item selection task (**D**) in each group. * *p* < 0.05, ** *p* < 0.01 vs. PE group. # *p* < 0.05, ## *p* < 0.01 vs. C group.

To explore the influence of 11 weeks of physical exercise interventions on the EFs of the children, a mixed variance design of 2 × 2 repeated measures ANOVA was used with the within factor (before/after intervention) and the between factor EXERCISE (exercise/no exercise). Physical exercise was used as the inter-subject variable (Accuracy Working memory span: F(1, 87) = 22.09; Accuracy The simple reaction test: F(1, 87) = 49.53; Accuracy Go/No go: F(1, 87) = 15.69; Accuracy Flexible item selection task: F(1, 87) = 103.22. Time was used as the internal variable of the subject (Accuracy Working memory span: F(1, 87) = 22.09; Accuracy The simple reaction test: F(1, 87) = 88.05; Accuracy Go/No go: F(1, 87) = 26.27; Accuracy Flexible item selection task: F(1, 87) = 62.83). Physical exercise x time had an interactive effect (Accuracy Working memory span: F(1, 87) = 25.21; Accuracy The simple reaction test: F(1, 87) = 44.88; Accuracy Go/No go: F(1, 87) = 12.76; Accuracy Flexible item selection task: F(1, 87) = 61.27). We observed the changes in the EFs parameters, which are presented in Figure 4. After 11 weeks of physical exercise interventions, the EFs parameters in the PE group were significantly improved than those before the 11 weeks (*p* < 0.05, *p* < 0.01), but there were no significant differences in the C group (*p* > 0.05). In addition, significant differences were observed in the post-test EFs parameter results between the C and PE groups after physical exercise interventions (*p* < 0.05, *p* < 0.01). These results indicate that 11 weeks of physical exercise interventions improved EFs parameters in children.

## 4. Discussion

The aim of our study was to reveal the effects of physical exercise interventions on the PF and EFs of six-year-old Chinese elementary school children for the duration of 11 weeks. The body composition, PF and EFs parameters were measured before and after the 11-weeks physical exercise interventions or control condition. The present findings support the effectiveness of physical exercise and expanded previous research in several important ways. Moreover, the design of this study allowed us to obtain a more complete picture of the effect of physical exercise on PF and EFs. Physical fitness and cognitive development in children are influenced by various sport and play activities. The three measurement results showed physical exercise improved the PF and EFs of the children. The following results will be discussed.

Body composition parameters in childhood can have lifelong consequences on terms overall health, academic performance, and work ability [27]. In this study, we first investigated the children’s body composition. To this end, six-year-old children were classified (PE = 43, C = 46) to identify the differences in body composition, and to explore the effect of physical exercise on body composition in children. The results of our study demonstrated no statistically significant difference of body composition parameters between two groups before physical exercise interventions, including height, weight, BMI. Those phenomenon can also be demonstrated after 11 weeks physical exercise interventions. It is widely known that children are at a critical stage of growth and development; their height and weight changes accordingly with the developing of skeletal and muscular systems [28]. However, the body composition parameters in the C and PE groups also showed no statistically significant difference after the 11-week physical exercise interventions. Furthermore, the paired sample *t*-tests in our experiment also revealed that there was no statistically significant differences in the body composition parameters before and after the 11-week physical exercise interventions in each group. Contrastingly, Alberty et al. demonstrated that 24 months of physical exercise interventions (twice a week, 60 min per session) was associated with a significantly small increase in body composition parameters [29]. We presumed that 11 weeks of physical exercise in our study is not sufficient to observe changes in body composition. Thus, we concluded that there was no significant effect of physical exercise interventions on body composition in children.

Next, our study was conducted with the hypothesis that the PE group, compared with the C group, would have more positive effect in PF. Therefore, we examined the potential mechanisms of physical exercise-induced on PF in the children. Childhood is a critical stage for PF development, which is necessary for being physically active and achieving health-related benefits both in the short and long term [30]. Thus, long term exercise interventions inevitably have different degrees of positive influence on PF, such as muscle strength, flexibility, and VO2max [31]. Consistent with this study, we found that the PF parameters, including cardiopulmonary fitness, muscle strength, speed sensitivity, and flexibility quality, of the PE group significantly improved after the physical exercise training. Meanwhile, pre-test results in our study revealed that the baseline PF parameters were not statistically significantly different. This is consistent with our hypothesis, but the PF parameters were remarkably different between the two groups post-test. Moreover, our conclusion was later substantiated by Lee’s study. Consistent with a previous study, Lee’s article pointed out that 16 weeks of physical exercise interventions caused the greatest change in PF, which was demonstrated by the significant increase in PF variables, such as muscular strength, flexibility, muscular endurance, and balance showed a significant increase [32]. The latest research by Ortega et al. further investigated the effects of physical exercise on cardiorespiratory fitness among children who are overweight or obese. Their findings showed that cardiorespiratory fitness is improved among children aged 8 to 11 years who are overweight or obese after 20 weeks of exercise of relatively high intensity exercise for more than 1 h, 3 times per week [33]. These data directly supported our conclusion that 11 weeks of physical exercise interventions in our study improves PF parameters in children.

Last, EFs test was performed to examine the dynamic change in EFs parameters. Our study revealed that 11 weeks of physical exercise had significant effects on EFs parameters in children, including their inhibitory control, working memory, the reaction time, and cognitive flexibility. Several previous studies have tried to prove the positive impact of physical exercise interventions on children’s EFs. A meta-analysis demonstrated that a suitable exercise time and intensity can effectively improve the EFs among children and adolescents [34,35]. In addition, physical exercise has been reported to significantly change brain structure, which is critical for cognitive development including EFs [36,37]. Our study further showed that EFs including the inhibitory control, the working memory, the reaction time, and the cognitive flexibility of the PE group were significantly improved compared with that observed in the C group after 11 weeks physical exercise intervention. Similarly, after 11 weeks of physical exercise, the EF in the PE group had also improved. Many studies have shown that physical exercise can effectively improve people’s cognitive function [38,39], which is consistent with the results of this study that physical exercise can improve the EFs of children. Li L et al. provided neurological evidence for the moderating role of the left globus pallidus, which is a crucial structure for complex cognitive processing, in the positive effect of physical exercise on EFs, such as spatial learning and working memory. Increased left globus pallidus activity may be associated with the improvement of EFs due to its relation with the cerebral cortex and the thalamic neuron network [40]. Such evidence collectively indicates that 11 weeks of physical exercise interventions improve EFs parameters in children.

The advantage of our study is the comparison the EFs of children through physical exercise training, and the comparison of the changes of EFs after the intervention. However, our study also has some limitations, which need further discussion. First, we only tested four main EFs parameters (inhibitory control, working memory, the reaction time, and cognitive flexibility) in this study, but these parameters cannot completely evaluate the whole content of EFs. Second, we did not discuss whether the influence of the repeated measurements of the EFs test can be eliminated. Last, but most important, is that the effect of physical exercise on the EFs of children with obesity and other diseases needs to be studied further.

## 5. Conclusions

The results of our study demonstrated that 11 weeks of physical exercise can improve the PF and EFs of six-year-old children. Thus, physical exercise intervention can be considered as a safe and an economic choice for improving the PF and EFs in children.

## Figures and Tables

**Table 1:** Data of children’s basic information in each group (Mean ± SD).

	Pre-Test		Post-Test	
	C Group	PE Group	C Group	PE Group
Height [cm]	125.71 ± 2.09	125.29 ± 2.74	128.09 ± 2.76	127.47 ± 3.00
Weight [kg]	17.77 ± 2.70	18.91 ± 3.41	21.16 ± 3.44	22.20 ± 2.36
BMI [kg/m^2^]	17.21 ± 2.7	16.20 ± 1.77	17.11 ± 3.30	16.19 ± 2.21

## Data Availability

All original data in this manuscript could be obtained the first author.
